# Joint contractures responsive to immunosuppressive therapy in a girl with childhood‐onset systemic sclerosis double‐seropositive for rare anti‐nucleolar autoantibodies: a case report

**DOI:** 10.1186/s12969-021-00525-1

**Published:** 2021-03-20

**Authors:** Riki Tanaka, Yumi Tani, Yoichiro Kaburaki, Manao Kinoshita, Yasushi Kawaguchi, Yuka Okazaki, Masataka Kuwana, Masayoshi Harigai, Satoru Nagata, Takako Miyamae

**Affiliations:** 1 Department of Pediatrics, Tokyo Women’s Medical University, Tokyo, Japan, 8-1 Kawada-Cho, Shinjuku- Ku, 162-8666 Tokyo, Japan; 2 Institute of Rheumatology, Tokyo Women’s Medical University, Tokyo, Japan, 8-1 Kawada-Cho, Shinjuku- Ku, 162-8666 Tokyo, Japan; 3 Department of Dermatology, Faculty of Medicine, University of Yamanashi, Yamanashi, Japan, Shimokato, Chuo, 1110, 409-3898 Yamanashi, Japan; 4 Department of Allergy and Rheumatology, Nippon Medical School, 1-1-5 Sendagi, Bunkyo-Ku, 113- 8602 Tokyo, Japan

**Keywords:** Children, Joint contracture, Lung interstitial lesion, Systemic sclerosis, Anti Th/To, Anti-PM-Scl, Autoantibody

## Abstract

**Background:**

Systemic sclerosis (SSc; scleroderma) is an autoimmune connective tissue disease that affects the skin and subcutaneous tissue, in addition to the internal organs of the whole body. Onset in childhood is uncommon; however, both patients with childhood-onset and adult-onset SSc are positive for anti-nuclear antibodies (ANAs).Detection of SSc-related anti-nuclear antibodies is often useful for predicting clinical features, disease course, and outcomes.

**Case presentation:**

A 5-year-old Japanese female manifested gradually progressive abnormal gait disturbance, regression of motor development, Raynaud’s phenomenon, and the shiny appearance of the skin of the face and extremities at age 2. On admission, she presented a mask-like appearance, loss of wrinkles and skin folds, puffy fingers, moderate diffuse scleroderma (18/51 of the modified Rodnan total skin thickness score), and contracture in the ankle and proximal interphalangeal joints. Grossly visible capillary hemorrhage on nail fold and severe abnormal capillaroscopy findings including bleeding, giant loop and disappearance of capillaryconsistent with the late phase in SSc. A skin biopsy showed fibrous thickening of the dermis, entrapment of an eccrine sweat glands, and thickened fiber. Chest high-resolution computed tomographic scanning demonstrated patchy areas of ill-defined air-space opacity and consolidation predominantly involving the posterior basilar aspects of the lower lobes presenting withinterstitial lung disease. Positive ANA (1:160 nucleolar and homogeneous nuclear staining by indirect fluorescent antibody technique) and double-seropositive for anti-Th/To and anti-PM-Scl antibodies were identified. She was diagnosed with diffuse cutaneous SSc based on the Pediatric Rheumatology European Society/American College of Rheumatology/European League Against Rheumatism Provisional Classification Criteria for Juvenile Systemic Sclerosis and was successfully treated with immunosuppressive agents, including methylprednisolone pulses and intravenous cyclophosphamide.

**Conclusions:**

We experienced the first case of juvenile SSc with anti-PM-Scl and anti-Th/To antibodies. ILD was identified as a typical feature of patients with these autoantibodies; however, diffuse cutaneous SSc and joint contraction were uncharacteristically associated. The case showed unexpected clinical findings though the existence of SSc-related autoantibodies aids in determining possible organ involvement and to estimate the children’s outcome.

## Background

Systemic sclerosis (SSc; scleroderma) is a heterogeneous and rare autoimmune connective tissue disease that is characterized by immune dysregulation, extensive fibrosis, and vascular dysfunction [1, 2]. It affects internal organs such as the lungs, heart, gastrointestinal tract, and kidneys in addition to the skin and subcutaneous tissue. SSc is more common in women and the peak age of onset is between 20 and 50 years, although the disorder has been described in both young and elderly patients [3–5]. Onset in childhood is uncommon, and children younger than 10 years of age account for approximately 3 % of all cases, and patients between 10 and 20 years of age account for only 1.2–9 % [5]. Childhood onset occurs at a mean age of 8.1 years, and the peak age is between 10 and 16 years [6–8]. Diffuse cutaneous SSc (dcSSc) develops with equal frequency in boys and girls younger than 8 years old, whereas girls outnumber boys 3 to 1 for disease onset occurring in children older than 8 years [9]. Childhood-onset SSc, as compared with adulthood-onset SSc, is predominantly associated with dcSSc, calcinosis (*p* < 0.001), myositis (*p* = 0.050), and lower frequencies of internal organ involvement, such as interstitial lung disease (ILD, *p* = 0.050), pulmonary hypertension (*p* = 0.035), and esophageal involvement (*p* = 0.005) [10]. Anti-nuclear antibodies (ANAs) are frequently detected in patients with childhood-onset SSc as well as in those with adult-onset SSc. Although the role of ANA in the pathogenesis of SSc remains unclear, detection of SSc-related ANAs is useful for predicting clinical features, disease course, and outcomes. Herein, we report a girl with childhood-onset SSc positive for both anti-Th/To and anti-PM-Scl antibodies presenting prominent dcSSc with joint contractures and ILD.

## Case presentation

**Fig. 1:**
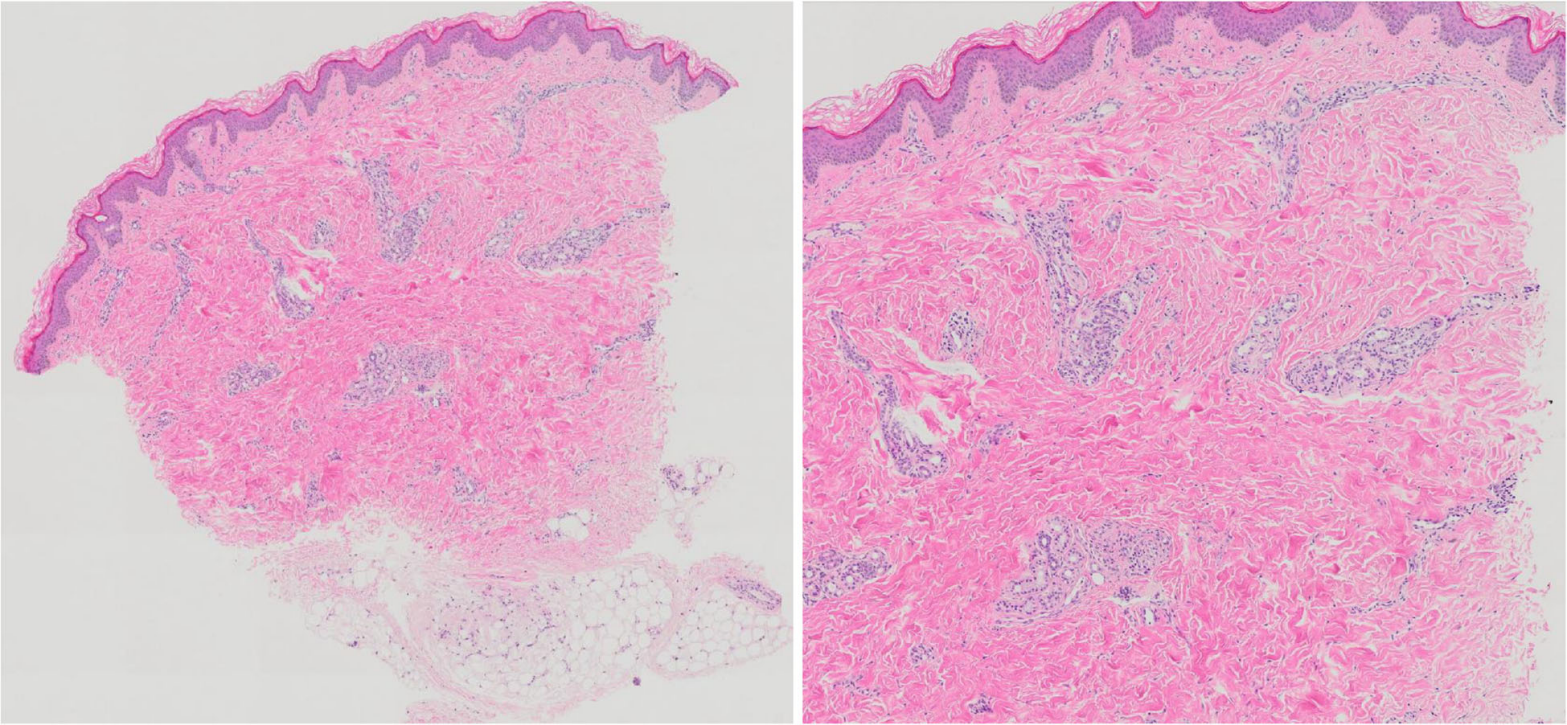
Histopathology findings in skin biopsy from the left dorsal pedis (low (left) and high (right) magnification); fibrous thickening of the dermis and relative entrapment of an eccrine sweat glands were observed. There were no pathological findings of vasculitis or vascular obstruction

**Fig. 2:**
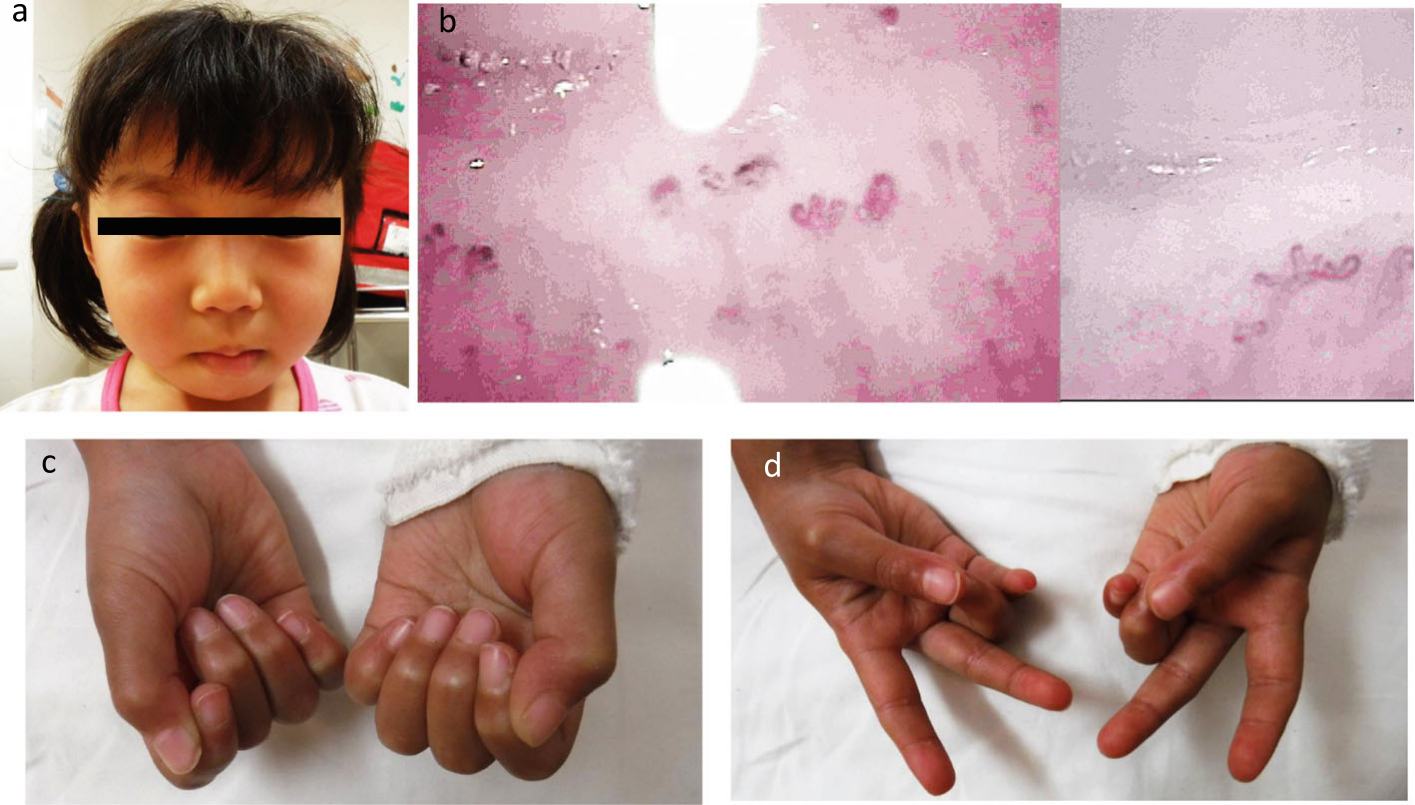
Clinical featured of the case patient. **a** Facial features at the time of admission; Shiny skin on the face. Face dimples previously could not be identified. **b** Nail fold capillaroscopy: severe abnormal capillaroscopy findings including bleeding, giant loop and disappearance of capillary consistent with the late phase in SSc. **c**, **d** Patient’s hands at the time of admission; she had puffy fingers and induration of the skin proximal to the *metacarpophalangeal joints*, the difficulty of grasping hands

**Fig. 3:**
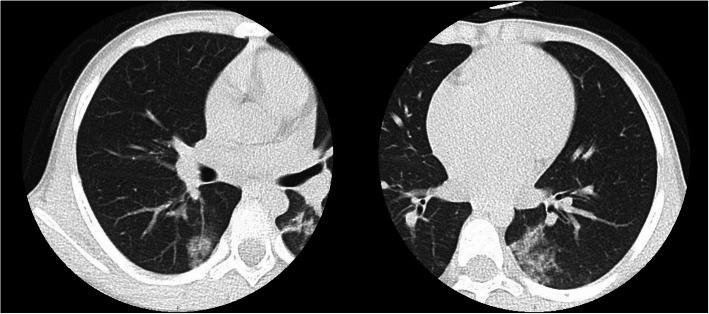
Chest high-resolution computed tomography (HRCT) scanning on admission. HRCT demonstrated patchy areas of ill-defined air-space opacity and consolidation predominantly involving the posterior basilar aspects of the lower lobes presenting with interstitial lung disease

**Fig. 4:**
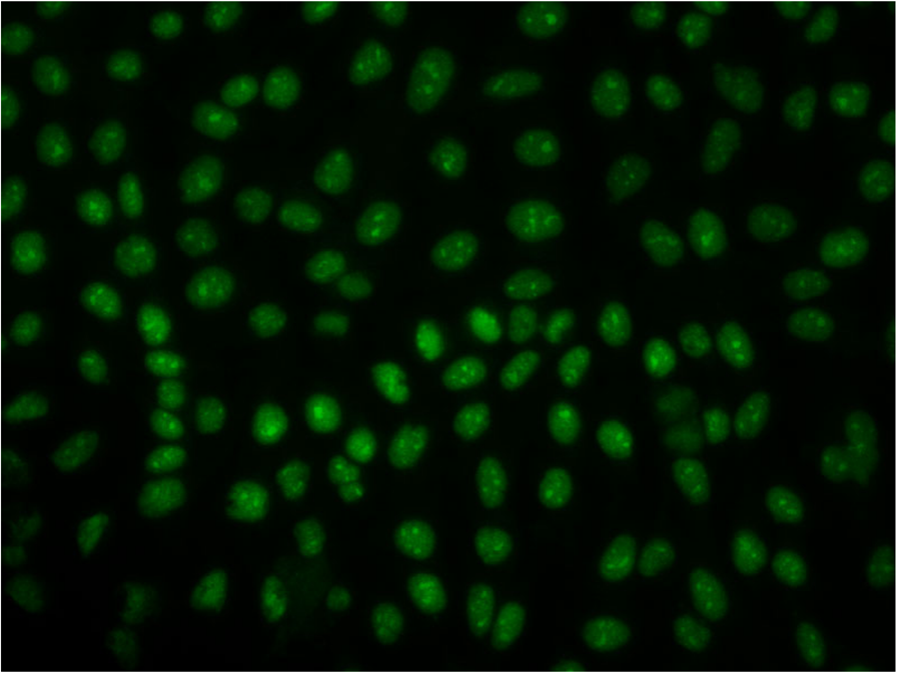
Anti-nuclear antibody was detected by indirect immunofluorescence. Nucleolar and homogeneous nuclear staining was identified at a serum dilution of 1:160

**Fig. 5:**
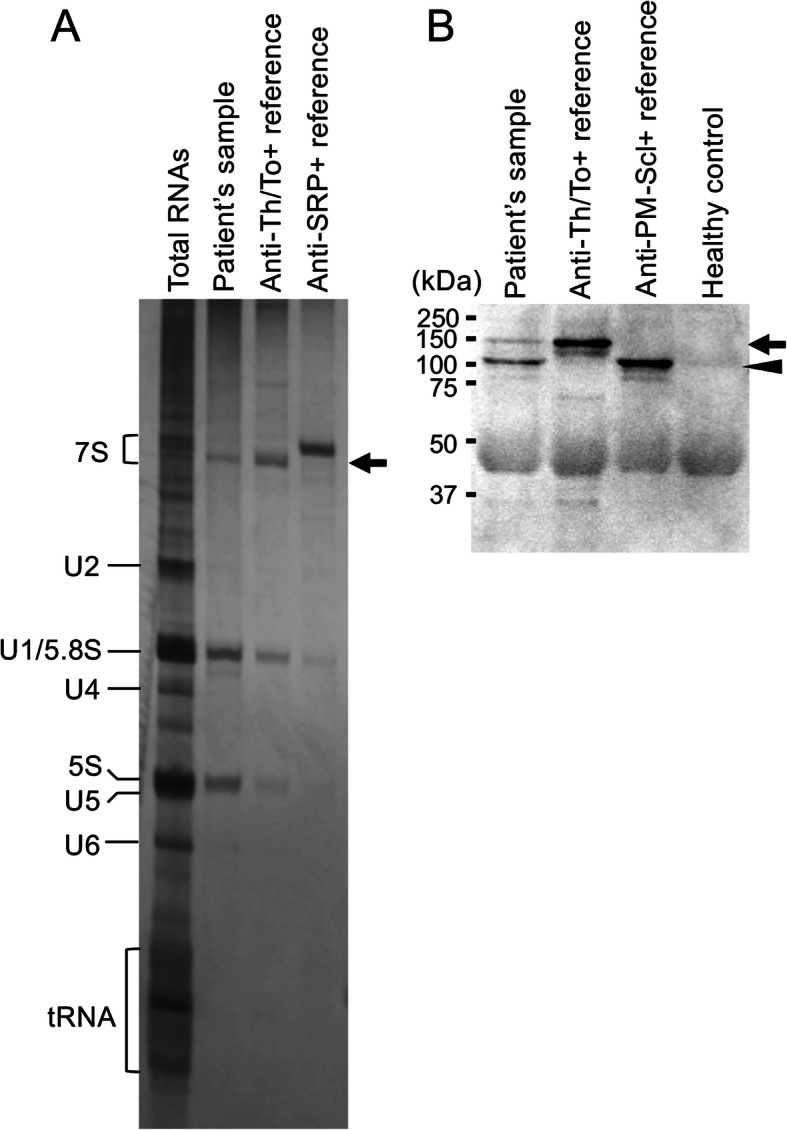
Identification of SSc-related autoantibodies. **a** RNA immunoprecipitation assay for our patient’s serum. The immunoprecipitates obtained by serum samples (our patient’s serum, anti-Th/To-positive reference serum, and anti-signal recognition particle [SRP]-positive reference serum) were subjected to urea-polyacrylamide gel electrophoresis, and the gels were stained with silver. Untreated total RNAs were also applied as references of cellular RNA components. An arrow indicates 7 − 2 RNA, a component of Th/To antigen. **b** Immunoprecipitation-immunoblot assay for our patient’s serum. The immunoprecipitates obtained by serum samples (our patient’s serum, anti-Th/To-positive reference serum, anti-PM-Scl-positive reference serum, and healthy control serum) were subjected to immunoblots probed with rabbit anti-hPOP1 and anti-PM-Scl-100 antibodies. Immunoreactive bands were visualized by a chemiluminescence detection system. An arrow indicates 120-kDa hPOP1 and an arrowhead indicates 100-kDa PM-Scl-100

**Fig. 6:**
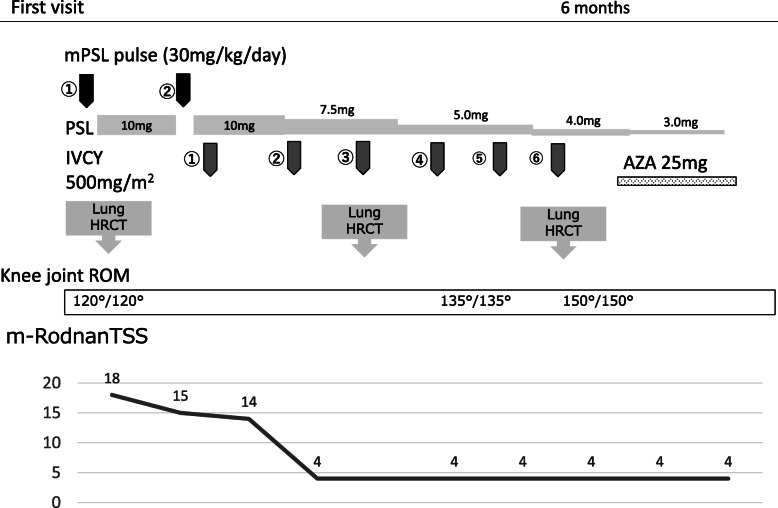
Clinical time course of the patient. PSL, prednisolone; MPT, methylprednisolone pulse; IVCY, intravenous cyclophosphamide; AZA, azathioprine; HRCT, high-resolution computed tomography; ROM, range of motion; m-Rodnan TSS, modified Rodnan total skin thickness score

The patient was a 5-year-old Japanese female, with no known relevant family history, showed gradually progressive abnormal gait disturbance, regression of motor development, Raynaud’s phenomenon, and the shiny appearance of the skin of the face and extremities at the age of 2. The skin abnormality became more obvious, and a skin biopsy was performed from the left dorsal pedis at a previous institution when she was 5 years old. It revealed a fibrous thickening of the dermis, relative entrapment of an eccrine sweat glands, and thickened collagenous fiber (Fig. 1). Based on the findings, she was referred to our hospital. She presented characteristic appearance with mask-like or mouse-facies as taut facial skin, loss of wrinkles and skin folds, puffy fingers with Raynaud’s phenomenon, and skin thickening of distal extremities beyond the elbows and knees (18/51 of the modified Rodnan total skin thickness score: mRSS) (Fig. 2) [11]. Grossly visible capillary hemorrhage on nail fold and severe abnormal capillaroscopy findings including bleeding, giant loop and disappearance of capillary consistent with the late phase in SSc. Contractures in the ankle and proximal interphalangeal joints resulted in gait disturbance and finger motion difficulty, respectively. There were neither abnormal neurological findings nor evidence suggesting myositis from information such as clinical muscle weakness, muscle derived enzyme, and muscle Magnetic Resonance Imaging at baseline intensive examination. Chest high-resolution computed tomography (HRCT) demonstrated patchy areas of ill-defined air-space opacity and consolidation predominantly involving the posterior basilar aspects of the lower lobes presenting with interstitial lung disease, although she had no symptoms suggesting respiratory abnormality, and no obvious finding could be detected by plain chest radiography (Fig. 3). Serum KL-6 level was 197 U/mL and was within the normal range. No abnormal findings were detected by electrocardiography or echocardiography. She manifested neither heartburn nor dysphagia, and findings of gastroesophageal reflux disease were not identified by esophagogastroduodenoscopy. Esophageal dilatation and/or dysmotility was not indicated by the upper gastrointestinal series. Blood examination showed positive ANA (nucleolar and homogeneous nuclear staining at a serum dilution of 1:160 by indirect immunofluorescence) (Fig. 4). The commercially available SSc-related autoantibodies, including anti-topoisomerase I (Scl-70), anticentromere, and anti-U1RNP, were not detected (Table 1). We then conducted an RNA immunoprecipitation assay and immunoprecipitation-immunoblot assay as previously described [12, 13]. The patient’s serum immunoprecipitated ribosomal RNAs and a 7 − 2 RNA that was consistent with the RNA component precipitated by a reference anti-Th/To-positive serum (Fig. 5). In addition, the immunoprecipitation-immunoblot assay probed with anti-hPOP1 and anti-PM-Scl-100 antibodies revealed that the patient’s serum contained both anti-Th/To and anti-PM-Scl antibodies (Table 1). The patient was diagnosed with diffuse cutaneous SSc, based on the Pediatric Rheumatology European Society/American College of Rheumatology/European League Against Rheumatism Provisional Classification Criteria for Juvenile Systemic Sclerosis [14]. She was treated with 2 courses of methylprednisolone pulse therapy (30 mg/kg/day for 3 days each course) followed by 10 mg/day of oral prednisolone (PSL) (Fig. 6). Subsequently, 6 courses of monthly intravenous cyclophosphamide (IVCY, 500 mg/m^2^ each course) therapy were administered. In the second course of IVCY, her skin thickening improved and the mRSS was 4/51. Just before the third course of IVCY, the interstitial lesions at the basal lung field were not identifiable on follow-up HRCT, and joint contracture also improved. After completing 6 courses of IVCY without major adverse events, she was maintained with 25 mg/day of azathioprine and PSL. Her PSL dose was reduced from 10 mg/day to 3 mg/day during 7 months of the time course.

**Table 1:** SSc-related autoantibodies

SSc-related autoantibodies	Measurement methods	Present Case
Antinuclear antibodies (ANA)	Indirect immunofluorescence	1:160 nucleolar and homogeneous nuclear
Anti-topoisomerase I (anti-Scl70)	Chemiluminescent enzyme immunoassay (commercially available)	negative
Anticentromere	Enzyme-linked immunosorbent assay (commercially available)	negative
Anti-RNA polymerase III	Enzyme-linked immunosorbent assay (commercially available)	negative
Anti-U1RNP	Chemiluminescent enzyme immunoassay (commercially available)	negative
Anti-Th/To	Immunoprecipitation-immunoblot assay	positive
Anti-PM-Scl	Immunoprecipitation-immunoblot assay	positive

## Discussion

In this report, we describe a case of early childhood-onset SS. Particularly notable findings were the simultaneous identification of SSc-related anti-Th/To and anti-PM-Scl autoantibodies, rare anti-nucleolar autoantibodies. ANA seropositivity in three large pediatric SSc series was 78–97 %, which is lower in children than in adults [6, 8, 9]. The predominant ANA patterns were speckled and nucleolar staining. The pathogenesis of this disorder is not fully understood, but growing evidence suggests that T cells, B cells, and SSc-related autoantibodies participate in the disease process [15, 16]. It has been reported that over 85 % of SSc patients have SSc-related autoantibodies, and that their detection is useful in diagnosis and disease subtyping [17, 18]. In adult-onset SSc, anti-topoisomerase I, anti-centromere, and anti-RNA polymerase III are the most prevalent autoantibodies [18]. Anti-topoisomerase I is associated with dcSSc and ILD, and is the most common autoantibody in childhood-onset SSc patients (28–34 %) [6, 8, 9]. Anti-RNA polymerase III is associated with dcSSc and the greatest risk for developing scleroderma renal crisis [19], and is rarely found in juvenile-onset SSc. Patients with juvenile-onset SSc more frequently had an overlap syndrome of lcSSc and myositis and showed a higher prevalence of autoantibodies associated with sclerodermatomyositis compared with adult-onset SSc, that is, anti-PM-Scl (14 % vs. 3 %; *p* < 0.0001) and anti-U1RNP antibodies (16 % vs. 7 % in adults; *p* < 0.0001) [6]. Anti-PM-Scl antibody predominantly represents nucleolar staining by indirect immunofluorescence, often with a moderately high ANA titer [20]. In a large cohort of patients with anti-PM-Scl antibody, ILD was identified in 13 % of patients with this antibody alone, and in 29 % of patients with this and other SSc-related autoantibodies, including anticentromere, anti-topoisomerase I, and/or anti-RNA polymerase III [21]. Anti-Th/To antibody is another anti-nucleolar antibody and occurs in less than 5 % of SSc patients in adults, but not in children [6]. Almost all patients with anti-Th/To antibody have lcSSc and are associated with either interstitial lung lesions (ILD) (45 %) or pulmonary arterial hypertension (25 %), within adult patients with lcSSc [22]. In a Japanese report of diffuse cutaneous SSc with anti-Th/To antibody, all three patients presented ILD and coexistence of anti-topoisomerase I antibody [23]. This is the first case report of juvenile SSc with two rare anti-nucleolar specificities, including anti-PM-Scl and anti-Th/To antibodies. Notably, patients with childhood-onset SSc had a significantly higher positivity for two or more SSc-related autoantibodies (8 % vs. 1.5 %; *p* < 0.005) than adult-onset patients [6]. Our patient had dcSSc with prominent joint contractures was apparently inconsistent with the clinical features of anti-PM-Scl or anti-Th/To antibodies reported in adults. Clinical characteristics associated with SSc-related antibodies are derived from studies almost exclusively involving adult-onset SSc patients, which might differ between juvenile and adult patients with SSc.

## Conclusions 

We experienced the first known case of juvenile SSc with anti-PM-Scl and anti-Th/To antibodies. ILD was identified as a typical feature of the autoantibodies; however, diffuse cutaneous SSc and joint contraction were uncharacteristically associated. The case revealed unexpected clinical findings though the existence of SSc-related autoantibodies basically aided in determining of possible organ involvement and estimation of the patient’s outcome.

## Abbreviations

SSc: Systemic sclerosis
dcSSc: Diffuse cutaneous SSc
ILD: Interstitial lung disease
mRSS: Modified Rodnan total skin thickness score
ANA: Anti-nuclear antibody
HRCT: Chest high resolution computed tomographic scanning
PSL: Prednisolone
IVCY: Intravenous cyclophosphamide

## Declarations

### Availability of data and materials

The datasets generated and/or analysed during the current study are not publicly available due [REASON WHY DATA ARE NOT PUBLIC] but are available from the corresponding author on reasonable request.
